# Mosquitoes Inoculate High Doses of West Nile Virus as They Probe and Feed on Live Hosts

**DOI:** 10.1371/journal.ppat.0030132

**Published:** 2007-09-14

**Authors:** Linda M Styer, Kim A Kent, Rebecca G Albright, Corey J Bennett, Laura D Kramer, Kristen A Bernard

**Affiliations:** 1 Arbovirus Laboratories, Wadsworth Center, New York State Department of Health, Slingerlands, New York, United States of America; 2 Department of Biomedical Sciences, School of Public Health, University at Albany, Albany, New York, United States of America; The Scripps Research Institute, United States of America

## Abstract

West Nile virus (WNV) is transmitted to vertebrate hosts by mosquitoes as they take a blood meal. The amount of WNV inoculated by mosquitoes as they feed on a live host is not known. Previous estimates of the amount of WNV inoculated by mosquitoes (10^1.2^–10^4.3^ PFU) were based on in vitro assays that do not allow mosquitoes to probe or feed naturally. Here, we developed an in vivo assay to determine the amount of WNV inoculated by mosquitoes as they probe and feed on peripheral tissues of a mouse or chick. Using our assay, we recovered approximately one-third of a known amount of virus inoculated into mouse tissues. Accounting for unrecovered virus, mean and median doses of WNV inoculated by four mosquito species were 10^4.3^ PFU and 10^5.0^ PFU for Culex tarsalis, 10^5.9^ PFU and 10^6.1^ PFU for *Cx. pipiens*, 10^4.7^ PFU and 10^4.7^ PFU for *Aedes japonicus,* and 10^3.6^ PFU and 10^3.4^ PFU for *Ae. triseriatus*. In a direct comparison, in vivo estimates of the viral dose inoculated by *Cx. tarsalis* were approximately 600 times greater than estimates obtained by an in vitro capillary tube transmission assay. Virus did not disperse rapidly, as >99% of the virus was recovered from the section fed or probed upon by the mosquito. Furthermore, 76% (22/29) of mosquitoes inoculated a small amount of virus (∼10^2^ PFU) directly into the blood while feeding. Direct introduction of virus into the blood may alter viral tropism, lead to earlier development of viremia, and cause low rates of infection in co-feeding mosquitoes. Our data demonstrate that mosquitoes inoculate high doses of WNV extravascularly and low doses intravascularly while probing and feeding on a live host. Accurate estimates of the viral dose inoculated by mosquitoes are critical in order to administer appropriate inoculation doses to animals in vaccine, host competence, and pathogenesis studies.

## Author Summary

Since it was first introduced into the United States in 1999, West Nile virus (WNV) has caused significant disease in humans, horses, and other animals. WNV is transmitted to humans and other vertebrate hosts by female mosquitoes as they take a blood meal. Currently, the amount of virus inoculated by mosquitoes while feeding on live hosts is unknown, and accurate estimates are critical so that appropriate challenge doses can be used in vaccine and viral pathogenesis studies. Here, we use a novel technique to determine the dose of WNV inoculated by mosquitoes as they probe and feed on the peripheral tissues of live animals. We found that mosquitoes inoculate high doses of virus into host tissues; these doses are 10 to 1,000 times higher than previous estimates obtained with assays that do not allow mosquitoes to probe or feed naturally. We also found that mosquitoes inoculate low doses of virus directly into the blood while blood feeding. Direct introduction of virus into the blood may alter viral tropism and cause low rates of infection in co-feeding mosquitoes. Our study provides new insights into the transmission of an emerging viral pathogen and the interaction of virus with its mosquito vector and vertebrate host.

## Introduction

West Nile virus (WNV) has become the most prevalent arbovirus in the United States, causing more than 24,000 reported human cases and 960 deaths since it was first detected in New York in 1999 [1]. The virus is maintained in an enzootic cycle involving birds and mosquitoes (primarily *Culex* species) [2]. Most humans become infected with WNV through the bite of an infected mosquito. After locating a suitable host, a mosquito probes throughout the dermis with her mouthparts and imbibes blood once a blood vessel is pierced. Saliva (and virus, if a mosquito is infectious) is deposited into the host throughout the feeding process.

A fundamental component of the mosquito transmission process, namely how much virus mosquitoes inoculate into a host while feeding, is not known. Previous studies have used in vitro methods to estimate the WNV dose inoculated by mosquitoes. Depending on mosquito species, mean WNV titers ranged from 10^1.2^ to 10^4.3^ plaque forming units (PFU) [3–6]. Although in vitro methods are convenient and relatively easy to perform, they do not allow mosquitoes to probe or feed naturally. Most of the saliva deposited by mosquitoes while blood feeding is re-ingested [7,8]. Therefore, mosquitoes inoculate most of the saliva, and thus virus, during the probing phase. Because in vitro techniques do not allow mosquitoes to probe naturally, these techniques are likely to underestimate the dose of virus inoculated.

We developed an in vivo assay to determine the amount of WNV inoculated by mosquitoes as they probe and feed on peripheral tissues of a live host. We used this assay to determine the dose of WNV inoculated by two important enzootic vectors, *Cx. tarsalis* and *Cx. pipiens,* and two potential bridge vectors, *Ae. japonicus* and *Ae. triseriatus*, as they probed and fed on a mouse tail, mouse ear, or chick toe. In addition, we examined the movement of virus from the probing/feeding site, determined whether the amount of virus inoculated was related to mosquito probing time, compared in vitro and in vivo estimates of the dose of WNV inoculated by mosquitoes, and examined clearance of the virus from the blood of mice following intravenous inoculation. We found that mosquitoes inoculate high doses of WNV into hosts while probing and feeding, doses that are 10- to 1,000-fold higher than previous estimates. Additionally, we found that mosquitoes inoculate low amounts of WNV directly into the blood while feeding.

## Results

### Efficiency of Virus Recovery from Mouse Tissues

We determined our ability to recover a known amount of WNV (∼10^5^ PFU) inoculated subcutaneously into the tail and ear of three mice using our tissue grinding protocol (see Materials and Methods). As a control, the same volume of virus was inoculated directly into each of five microcentrifuge tubes. Control samples contained an average of 106,000 PFU, whereas mouse tails contained 30,900 PFU, and mouse ears contained 34,500 PFU. Assuming the same dose was inoculated into the ear and tail as was inoculated into the control samples, we recovered 29.2% of the inoculated virus from the mouse tail and 32.5% from the mouse ear. These results indicate that subsequent amounts of virus recovered from mouse tissues need to be multiplied by ∼3 to provide the actual amount of virus inoculated into these tissues by mosquitoes. Unless otherwise indicated, titers mentioned in the text or shown in figures are not adjusted for “unrecovered” virus.

### Amount of Virus Inoculated into Host Tissues by Mosquitoes

We determined the amount of virus inoculated by WNV-infected mosquitoes while probing and feeding on a mouse tail, mouse ear, or chick toe. Eight independent trials of this experiment were conducted, with orally infected or intrathoracically inoculated mosquitoes of four species (*Cx. tarsalis*, *Cx. pipiens*, *Ae. japonicus*, and *Ae. triseriatus*) (Table 1). *Cx. tarsalis* and *Cx. pipiens* are important enzootic vectors of WNV in the United States, and *Ae. japonicus* and *Ae. triseriatus* have been implicated as bridge vectors for WNV [9]. There was variation in the age of the hosts used in these studies and mosquito extrinsic incubation period following intrathoracic inoculation (Table 1); however, these variables did not have a significant effect on the amount of virus inoculated by mosquitoes (Spearman's rank correlation coefficient *p* > 0.05).

**Table 1:**
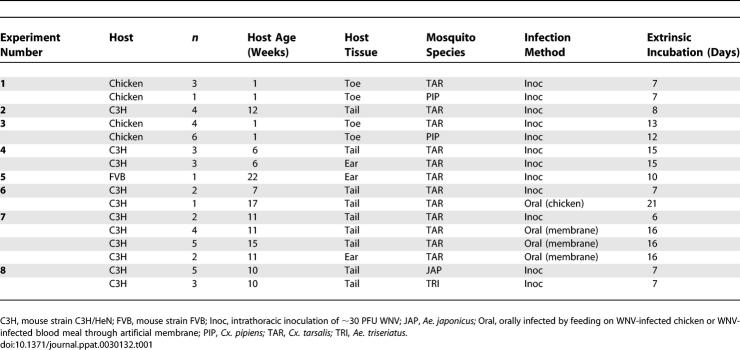
Details of Experiments to Determine the Dose of Virus Inoculated by Mosquitoes while Probing and Feeding on Live Hosts

**Figure 1:**
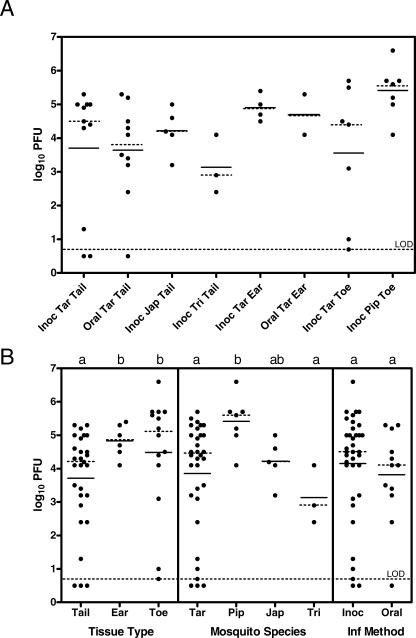
Mosquitoes Inoculate High Doses of WNV In Vivo under Various Experimental Conditions (A) WNV doses inoculated extravascularly by mosquitoes. *x*-Axis labels indicate mosquito infection method (Inoc = intrathoracic inoculation, Oral = orally infected), mosquito species (Tar = *Cx. tarsalis*, Pip = *Cx. pipiens*, Jap = *Ae. japonicus*, Tri = *Ae. triseriatus*), and tissue in which the mosquito probed or fed in (Tail = mouse tail, Ear = mouse ear, Toe = chick toe). Limit of detection (LOD) of plaque assay is shown. (B) Same data as in (A) but pooled by tissue type, mosquito species, and infection method. Within each larger grouping (tissue type, mosquito species, or infection method), groups designated with different lower case letters (above graph) are significantly different from one another (*p* < 0.05 by Mann-Whitney or Kruskal-Wallis tests). Solid line shows mean titer, and dashed line shows median titer, for each group.

Mosquitoes inoculated high doses of WNV into host tissues while probing and feeding (Figure 1A). The amount of virus recovered from tissues ranged from below the limit of detection (5 PFU) to 10^6.6^ PFU. Mean and median values of the groups ranged from 10^2.9^ PFU to 10^5.5^ PFU. Although there was variation in mean and median inoculated doses among the groups, these differences were not statistically significant (Kruskal-Wallis test, *p* > 0.05) (Figure 1A). Mosquito infection method (orally infected or intrathoracically inoculated) also had no effect on the amount of virus recovered from tissues when the analysis was restricted to mouse tissues fed upon by *Cx. tarsalis* (Wilcoxon test, *p* > 0.5) or when the data were pooled (Figure 1B). Similarly, the type of host tissue fed upon by *Cx. tarsalis* (mouse tail, mouse ear, or chick toe) had no effect on inoculation dose (Kruskal-Wallis test, *p* = 0.46). However, when the data were pooled, more WNV was recovered from the mouse ear and chick toe than from the mouse tail (Kruskal-Wallis test, *p* = 0.03) (Figure 1B). The amount of virus recovered also varied by mosquito species; *Cx. pipiens* feeding on chick toes inoculated higher amounts of virus than did *Cx. tarsalis* feeding on chick toes (ANOVA of ranked data, *p* = 0.04). When the data were pooled, *Cx. pipiens* inoculated higher doses than did *Cx. tarsalis* or *Ae. triseriatus* (Kruskal-Wallis test, *p* = 0.01). Mean and median doses inoculated by *Cx. pipiens* were 10^5.4^ and 10^5.6^ PFU, *Cx. tarsalis* inoculated mean and median doses of 10^3.8^ and 10^4.5^ PFU, *Ae. japonicus* inoculated mean and median doses of 10^4.2^ and 10^4.2^ PFU, and *Ae. triseriatus* inoculated mean and median doses of 10^3.1^ and 10^2.9^ PFU. Considering that we recovered approximately one-third of the virus from mouse tissues, and assuming that we would have recovered a similar proportion from chick tissues, these mean and median values should represent the minimum average values inoculated by mosquitoes. Adjustment of these values to account for unrecovered virus suggests that *Cx. pipiens* inoculated mean and median doses of 10^5.9^ and 10^6.1^ PFU, *Cx. tarsalis* inoculated mean and median doses of 10^4.3^ and 10^5.0^ PFU, *Ae. japonicus* inoculated mean and median doses of 10^4.7^ and 10^4.7^ PFU, and *Ae. triseriatus* inoculated mean and median doses of 10^3.6^ and 10^3.4^ PFU.

### Spread of Virus following Inoculation by Mosquitoes

**Figure 2:**
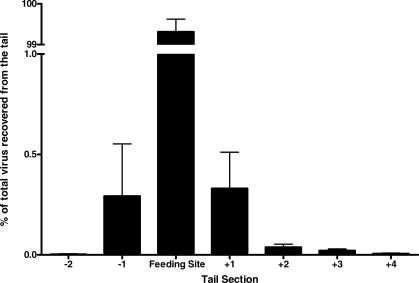
Movement of Virus from Mosquito Feeding Site on Tail Most of the virus inoculated by mosquitoes was recovered from the tail section(s) that the mosquito probed and fed in (feeding site). Percent of total virus recovered from each tail section. Data from each mouse tail were shifted to align feeding sites. If mosquitoes probed or fed in two sections, those sections were summed and combined into one feeding site. Tail sections toward the tail tip in relation to the feeding site are labeled −1 and −2. Tail sections toward the body in relation to the feeding site are labeled +1 to +4. Includes data from orally and parenterally infected *Cx. tarsalis*, *Ae. japonicus*, and *Ae. triseriatus* feeding on a mouse tail.

To investigate virus spread through the tail, we divided it into 1.0-cm sections following mosquito feeding and determined the amount of virus in each section. Almost all of the virus (99.3%) was recovered from the section of tail that the mosquito probed or fed in, suggesting that virus does not disperse very quickly from the site of inoculation (Figure 2). Most of the remaining virus (0.6%) was recovered from tail sections on either side of the feeding site.

**Figure 3:**
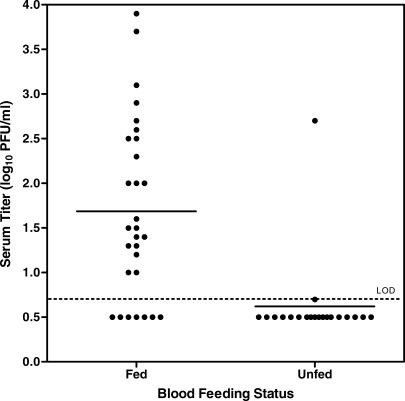
Mosquitoes Inoculate a Small Amount of Virus Intravascularly when Blood Feeding Amount of WNV in the serum of animals following mosquito feeding on distal tissues. Mosquitoes either probed tissue and imbibed blood (Fed) or probed without blood feeding (Unfed). Solid line indicates mean WNV titer in serum of each group. LOD of plaque assay is shown.

Within 5 minutes after the cessation of mosquito feeding, we collected a serum sample from the heart (mouse) or wing vein (chick) to determine if virus had entered the blood. A small amount of virus (mean: 10^2.0^, range: 10^0.7^–10^3.9^ PFU/ml) was recovered from the sera of 24 out of 49 animals (49%) (Figure 3). Interestingly, there was a strong relationship between mosquito blood feeding status (blood fed or unfed) and the presence of an early viremia. Mosquitoes imbibed blood from 29 out of 49 animals (59%), and an early viremia was detected in 76% (22 out of 29) of these animals. Only two of 20 animals (10%) developed an early viremia in the absence of mosquito blood feeding. An analysis showed that blood feeding was significantly associated with development of an early low viremia (χ^2^ = 20.55, *p* < 0.0001), suggesting that mosquitoes inoculate a small amount of virus directly into the host's blood while feeding. The detection of early viremia in two animals on which mosquitoes had not blood fed could have been due to misclassification of the mosquito feeding status (i.e., the mosquito did blood feed, but it was not detected) or to virus quickly entering the blood directly from the tissue.

### Effect of Probing Time on Amount of Virus Inoculated by Mosquitoes

**Figure 4:**
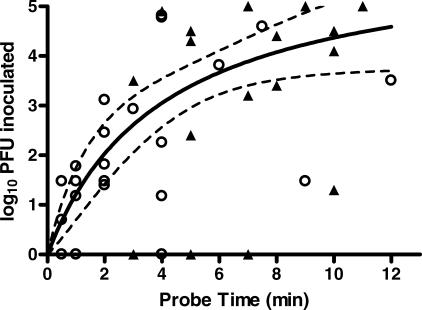
Amount of WNV Inoculated by Mosquitoes into Mouse Tails Increases with Probing Time Included on graph are data from the probe time experiment (open circles) and from other experiments (filled triangles) in which parenterally or orally infected *Cx. tarsalis* probed and fed on a mouse tail. Solid line shows best-fit one-site binding model, and dotted lines show 95% confidence band of the best-fit curve. LOD of plaque assay was 5 PFU. Values less than the LOD are reported as 0.

Although mosquito blood feeding status impacted the development of an early viremia, it had no effect on the dose of virus recovered from host tissues (Mann-Whitney test, *p* = 0.57). Therefore, we determined if the other component of mosquito feeding (probing) impacted the virus dose inoculated by mosquitoes. In the above described experiments, average probing times of *Cx. tarsalis* were 5–7 min, *Cx. pipiens* probed for 4 min, and *Ae. triseriatus* and *Ae. japonicus* probed for 2 and 3 min, respectively (Tables S1–S7). To further investigate the effect of shorter probing times on the amount of WNV inoculated, we allowed parenterally infected *Cx. tarsalis* to probe a mouse tail for 0.5 min, 1 min, 2 min, 4 min, or the maximum time. We combined the results of this experiment with the results of our previous experiments in which orally and parenterally infected *Cx. tarsalis* had probed and fed on mouse tails. A one-site binding model was fitted to the data (y = 5.402 * x / (3.551 + x)) using GraphPad Prism 4.0 (R^2^ = 0.4019) (Figure 4). This model suggests that inoculated doses increase rapidly with longer probing times, up to 4–6 min. Beyond this point, doses increase more slowly or plateau. The amount of WNV inoculated by *Cx. tarsalis* levels out at 10^3.5^–10^4.5^ PFU, or 10^4.0^–10^5.0^ PFU when quantities are adjusted for unrecovered virus. We did not see any relationship between probing time and inoculation dose for field populations of *Ae. japonicus* or *Ae. triseriatus* (linear regression: R^2^ = 0.10, *p* = 0.45) (Tables S6 and S7), suggesting that the effect of probe time on inoculation dose may be species specific or may be related to colonization status.

### In Vivo and In Vitro Estimates of Viral Dose Inoculated by Mosquitoes

**Figure 5:**
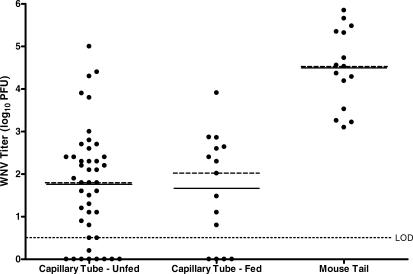
Mosquitoes Inoculate Higher WNV Doses into Mouse Tails than into Glass Capillary Tubes Intrathoracically inoculated *Cx. tarsalis* females (*n* = 15) probed/fed on a mouse tail; the tail was removed and macerated, and WNV titer was determined (Mouse Tail). Salivary secretions were collected 2–4 h later from these same mosquitoes by an in vitro capillary tube transmission assay (Capillary Tube – Fed). At the same time, salivary secretions were collected from intrathoracically inoculated female *Cx. tarsalis* from the same cohort that had not fed on a mouse tail (*n* = 44) (Capillary Tube – Unfed). Shown are the combined data from two independent studies. Mean and median WNV titers in each column are designated by solid and dashed horizontal lines. LOD of plaque assay is shown.

We compared in vivo and in vitro estimates of viral dose inoculated by allowing *Cx. tarsalis* females to feed on a mouse tail, and then within 2–4 h allowing those same mosquitoes to salivate into a capillary tube. The amount of virus recovered from the tail (mean = 10^4.5^ PFU) was ∼600-fold higher than that recovered from the capillary tube (mean = 10^1.7^ PFU) (paired *t*-test: *t* = 7.96, *df* = 14, *p* < 0.0001) (Figure 5). Low amounts of virus in the capillary tube assay were not explicable by the recent blood feeding by mosquitoes, because mosquitoes that had not taken a blood meal secreted a similar amount of virus (mean = 10^1.8^ PFU) (*t*-test: *t* = 0.25, *df* = 57, *p* = 0.81).

### WNV Titer in Mosquito Body Segments

**Figure 6:**
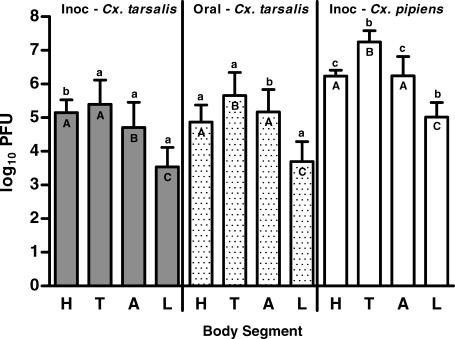
*Culex* Mosquitoes Contain High Amounts of WNV Geometric mean WNV titers in head (H), thorax (T), abdomen (A), and legs (L) of orally (Oral) or parenterally (Inoc) infected *Cx. tarsalis* and parenterally infected *Cx. pipiens*. Orally infected mosquitoes were tested at day 16 PI and parenterally infected mosquitoes were tested at day 7 PI. Letters within bars designate body segment titers that are significantly different from one another (*p* < 0.05) within each mosquito group. Letters above bars designate the mosquito groups that differ significantly from one another (*p* < 0.05) when compared by body segment. Error bars show standard deviation. LOD of plaque assay was 5 PFU.

We measured the amount of WNV in the body segments of parenterally infected *Cx. tarsalis*, orally infected *Cx. tarsalis*, and parenterally infected *Cx. pipiens* to investigate virus distribution within the mosquito body, and to compare doses inoculated into mouse and chick tissues with the amount of virus in the thorax (where the salivary glands are located) (Figure 6). Within all three groups, the thorax had the highest, and the legs had the lowest, viral loads (ANOVA, *p* < 0.05). In pairwise comparisons by body segment among the three groups, *Cx. pipiens* females had higher WNV loads in each body segment than did either group of *Cx. tarsalis* (ANOVA, *p* < 0.05). The amounts of virus in the thorax and legs of orally and parenterally infected *Cx. tarsalis* were not statistically different from one another; however, viral loads were 3-fold higher in the abdomen and 2-fold lower in the head in orally infected *Cx. tarsalis* compared to parenterally infected *Cx. tarsalis* (ANOVA, *p* < 0.05). High viral loads found in *Cx. pipiens* body segments corresponded to the high doses recovered from chick toes fed upon by this species. Average viral loads in the thorax were 10^5.4^ PFU for parenterally infected *Cx. tarsalis*, 10^5.7^ PFU for orally infected *Cx. tarsalis*, and 10^7.3^ for parenterally infected *Cx. pipiens*. Using these data along with the adjusted inoculation doses, we estimate that *Cx. tarsalis* inoculates 4%–8% and *Cx. pipiens* inoculates 4% of the total amount of virus in the thorax into host animal tissues while probing and feeding.

### Clearance of Virus from Blood following Intravenous Inoculation

**Figure 7:**
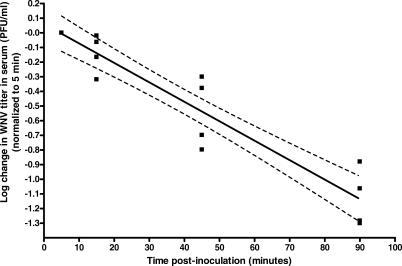
Clearance of WNV from the Serum following Intravenous Inoculation Mice (*n* = 4) were inoculated intravenously with 10^5^ PFU and serum samples taken at various times PI. For each mouse, WNV titers in the serum were normalized to the 5-min serum titer and reported as log change in WNV serum titer. Solid line indicates best-fit linear regression (R^2^ = 0.90, *p* < 0.0001); dashed lines indicate 95% confidence band for regression line.

Mice were inoculated intravenously with 10^5^ PFU into the lateral tail vein, and serum samples were taken at various times post-inoculation (PI) to investigate virus clearance rates from the blood. Virus titers in the blood decreased in a linear manner and were still detectable at 90 min PI. Rates of viral clearance were obtained from a linear regression model that was fit to the data (R^2^ = 0.90, *p* < 0.0001) (Figure 7). Virus titers in the blood decreased by 1 log_10_ PFU/ml in 79.8 min (95% CI: 66 to 96.7 min), and at 1 h after inoculation, virus titers decreased by 0.74 log_10_ PFU/ml (95% CI: −0.84 to −0.64 log_10_ PFU/ml).

## Discussion

We have developed an in vivo assay to determine the amount of WNV that mosquitoes inoculate while feeding on a live host. We used two important enzootic vectors for WNV in the United States, *Cx. tarsalis* and *Cx. pipiens* [9]. In addition, we used two *Aedes* species, *Ae. triseriatus* and *Ae. japonicus*. WNV has been isolated from field specimens of both *Aedes* species [10]. In addition, both are competent vectors in the laboratory [9,11] and have been implicated as possible bridge vectors (i.e., transmission from the avian enzootic cycle to humans) [9]. We selected host feeding sites that would allow removal and assay of the entire tissue, i.e., mouse tail, mouse ear, and chick toe, and determined the efficiency of viral recovery. Correcting for unrecovered virus, we estimate that *Cx. tarsalis* inoculated mean and median doses of 10^4.3^ and 10^5.0^ PFU, *Cx. pipiens* inoculated mean and median doses of 10^5.9^ and 10^6.1^ PFU, *Ae. japonicus* inoculated mean and median doses of 10^4.7^ and 10^4.7^ PFU, and *Ae. triseriatus* inoculated mean and median doses of 10^3.6^ and 10^3.4^ PFU.

These doses are 10- to 1,000-fold higher than in vitro estimates of WNV inoculated by *Cx. tarsalis* (∼10^2.0^), *Cx. pipiens* (10^2.3^–10^2.8^), and *Cx. pipiens quinquefasciatus* (10^4.3^ PFU) [3–6]. In a direct comparison between the two assays with *Cx. tarsalis*, we found that WNV titers obtained by the in vivo tail assay were 600-fold higher than titers from the in vitro capillary tube assay. This result differs from the findings of a similar study with Venezuelan equine encephalitis virus (VEEV) in which more virus was recovered from capillary tube transmission assays than from mouse tails [12]. Another unique feature of our data was the consistently high doses recovered from tissues, resulting in median doses that were as much as eight times higher than mean doses. Previous studies using the capillary tube assay had shown the opposite result: median doses were lower than mean doses [4–6].

For our in vivo assay, we estimated that we were able to recover approximately one-third of a known amount of virus that had been inoculated by needle into mouse tissues. Incomplete recovery was most likely due to several factors. First, virus would have begun to enter host cells during the time between virus inoculation and tissue harvesting/freezing. Although this time was short (<20 min), it is likely that some of the virus became unmeasurable by plaque assay after viral entry. Another possible factor is trapping of virus in the tissue as a result of incomplete maceration and homogenization. In this case, the virus would be pelleted along with the tissue during centrifugation. In a similar mouse tail experiment, “nearly all” of the injected VEEV was recovered [12]. However, virus in that study was injected into the tail tip, which may have been easier to macerate, and lower doses (10–10^2.9^ PFU) were injected into the tissue, a fact which may have affected recovery [12].

Inoculated doses were not affected by the method of mosquito infection. Orally and parenterally infected *Cx. tarsalis* inoculated the same mean dose of virus into mouse tissues and also had similar viral loads in the thorax and legs. These results suggest that intrathoracic inoculation of mosquitoes with low WNV doses (30 PFU) mimics oral infection of mosquitoes in terms of viral yield in saliva. In contrast, Smith et al. [13] reported that parenterally infected *Ae. taeniorhynchus* and *Ae. albopictus* expelled lower amounts of VEEV into a capillary tube assay than did orally infected mosquitoes of the same species. The study by Smith et al. differs from ours in the virus, mosquito species, and in the intrathoracic inoculation dose (10^4^ PFU), any of which may account for the difference in results.

The amount of WNV inoculated by mosquitoes varied by mosquito species. *Cx. pipiens* inoculated on average 15- to 60-fold more virus than *Cx. tarsalis*. The maximum dose recovered from tissue fed upon by *Cx. pipiens* (10^6.6^ PFU) was more than 10-fold higher than the maximum dose recovered from tissue fed upon by *Cx. tarsalis* (10^5.4^ PFU). In addition, viral loads in the body segments of *Cx. pipiens* were significantly higher (10- to 100-fold) than viral loads in the body segments of *Cx. tarsalis*. *Ae. japonicus* inoculated WNV doses that were not significantly different from those inoculated by *Cx. tarsalis*, *Cx. pipiens,* and *Ae. triseriatus*. In contrast, *Ae. triseriatus* inoculated median WNV doses into the tail that were 400-fold lower than median doses inoculated by *Cx. pipiens*. In previous experiments, differences in the amount of virus expelled were observed for *Aedes* species: *Ae. taeniorhynchus* females expelled significantly more VEEV during an in vitro capillary tube assay than did *Ae. albopictus* females [13]. Additionally, several *Culex* species secreted significantly different doses of WNV during an in vitro capillary tube assay [5].

The amount of virus inoculated by mosquitoes generally increased with longer probing times, reaching a maximum dose after ∼4–6 min of probing. This result indicates that mosquitoes have the potential to inoculate a wide range of viral doses into vertebrate hosts, depending on how long they probe. Previous studies have shown that probing time varies by mosquito species and experimental conditions. Colonized *Cx. pipiens quinquefasciatus* probed for <1 min on chicks and >7 min on the shaved back of a mouse [14]. Studies with *Ae. aegypti* and Anopheles stephensi reported average probing times of <3 min [14–17]. *Culex* mosquitoes in our study probed for an average of 4–7 min, whereas *Aedes* mosquitoes probed for 2–3 min. The probing times that we observed could have been influenced by several factors. First, *Culex* mosquito colonies in our laboratory are blood fed using a membrane feeder rather than a natural bird host; consequently, there is no selection for mosquitoes that can quickly probe within tissue to find blood. In contrast, *Aedes* mosquitoes used in our experiments were collected from the field and had no exposure to artificial blood meals. Second, mosquitoes fed on anesthetized mice. Anesthesia has been shown to decrease cardiac function, causing reduced blood flow to the tissues. On the other hand, we observed similar probing times for mosquitoes feeding on chicks that were not anesthetized. Third, mosquitoes in our study fed on peripheral tissues (tail, ear, toe), which contain fewer blood vessels than do feeding locations toward the center of the body. Although such distal tissues may be less vascularized, mosquitoes, when given a choice, will naturally feed on these tissues (unpublished data). Finally, mosquitoes used in these studies were infected with WNV. WNV has been shown to cause severe cytopathology in salivary glands [18], which could lead to altered salivary gland function, and could ultimately increase the time needed for mosquitoes to find blood. We are not aware of studies that have quantified average probing times of *Culex* mosquitoes in the field to compare to our laboratory studies. Although there would be strong selection for fast feeding mosquitoes in the field (due to host defensive behavior), this same defensive behavior could lead to interrupted feedings, which would lengthen probing time and possibly expose multiple hosts [19].

Most of the virus (>99%) recovered from the mouse tail was recovered from the 1-cm section that the mosquito had probed or fed in. This result suggests that most virus is inoculated extravascularly while the mosquito is probing, and it further suggests that virus does not spread very quickly within the tissues. Extravascular inoculation of virus by mosquitoes has been demonstrated previously for Rift Valley fever virus, Saint Louis encephalitis virus, and VEEV [12,20,21].

Although mosquito inoculation of WNV is primarily extravascular, our results also indicate that some mosquitoes inoculate a small amount of virus directly into the blood while blood feeding. Virus (average titer = 10^2.0^ PFU/ml) was detected in the sera of 22 out of 29 animals when mosquitoes imbibed blood. However, when mosquitoes only probed and did not blood feed, virus was detected in the serum of only two of 20 animals. Direct inoculation of virus into the blood by mosquitoes could alter viral tropism and kinetics, and may explain the earlier development of viremia in hosts infected with WNV by mosquito bite compared to infection by needle inoculation [3].

In addition, our results suggest that the recent finding of non-viremic transmission of WNV by *Cx. pipiens quinquefasciatus* could have been due to infected mosquitoes inoculating a small amount of virus directly into the blood, which is imbibed by recipient mosquitoes, resulting in a low infection rate [22,23]. A recent publication supports this hypothesis. Low viremia levels (10^2.9^–10^4.2^ PFU/ml) were detected in house finches 30 to 45 min after infected mosquitoes fed, resulting in low infection rates in recipient mosquitoes [24]. At low viremia levels (<10^3^ PFU/ml), ∼60% of 5-μl blood meals taken by mosquitoes will contain no or <five virions, whereas ∼40% will contain >five virions, if virus is distributed at random in the blood [25]. Therefore, mosquito infection rates would be expected to be low (not zero), as long as some proportion of the mosquito population was able to become infected after ingesting low numbers of virions (i.e., a highly competent population). Additionally, our intravenous clearance study indicates that virus is cleared from the blood of mice at a rate of 0.7 log_10_ PFU/ml per hour. Therefore, 100 PFU of virus inoculated directly into the blood by a donor mosquito could theoretically circulate in the blood for 1–2 h and could infect recipient mosquitoes.

Most of the virus (50%–75%) in a mosquito was recovered from the thorax; amounts ranged from 10^3.7^ to 10^7.7^ PFU. The thorax contains not only musculature for locomotion, but also the salivary glands. Large aggregations of WNV virions were observed in salivary glands of orally infected mosquitoes at 14 d PI by electron microscopy [18,23]. Salivary glands of intrathoracically infected *Cx. pipiens quinquefasciatus* contained high titers of WNV (up to 10^7^ PFU equivalents) [23]. Assuming that much of the virus we detected in the thorax is contained within the salivary glands, thoracic viral load correlates well with the high doses inoculated into hosts by mosquitoes.

In conclusion, we found that mosquitoes inoculate high doses (10^4^–10^6^ PFU) of WNV extravascularly and low amounts (∼10^2^ PFU) intravascularly while probing and feeding on a live host. Direct inoculation of WNV into the host's blood during feeding may alter viral tropism, lead to earlier development of viremia, and result in infection of co-feeding mosquitoes. In a direct comparison, the amount of virus inoculated by a mosquito while feeding on a live host was ∼600-fold higher than that recovered during an in vitro capillary tube assay. These results suggest that the use of an in vitro capillary tube assay will result in lower estimates of the dose inoculated by mosquitoes and may also underestimate transmission rates (the proportion of mosquitoes that are capable of transmitting a pathogen once infected). Use of an accurate dose to infect animals is important in vaccine, host competence, and pathogenesis studies, especially because viral dose has been shown to affect WNV viremia and viral shedding [3].

## Materials and Methods

### Virus.

All experiments were conducted with WNV strain 3356 isolated in 2000 from the brain of a crow collected in Staten Island, New York [26]. This isolate was passed twice in Vero cells and had a titer of 10^9.5^ PFU/ml, as determined by plaque assay on Vero cells.

### Animals.

We used a *Cx. pipiens* colony established in 2004 from mosquitoes collected in Pennsylvania. We used the HVP *Cx. tarsalis* colony, which was derived from the WS colony, a colony that consisted of a mixture of field populations from California selected for high susceptibility to Western equine encephalitis (kindly provided by William Reisen, University of California, Davis). *Ae. japonicus* and *Ae. triseriatus* eggs were collected on expanded polystyrene floats [27] in Albany, New York, and reared in the laboratory at 22 °C. Emerged females were identified to species and used in our studies. Specific-pathogen-free Gallus gallus chicks (1–2 d old) were obtained from Charles River SPAFAS (http://www.criver.com/). Mouse strains C3H/HeN and C57/BL6 were obtained from Taconic Laboratories (http://www.taconic.com/) and strain FVB was obtained from Wadsworth Center, New York State Department of Health. All animals were housed in a BSL-3 animal facility. The use of chicks and mice in this experiment was approved and conducted in accordance with the Wadsworth Center Institutional Animal Care and Use Committee.

### Infection of mosquitoes with WNV.

Mosquitoes were infected with WNV by intrathoracic inoculation of ∼30 PFU WNV or by allowing mosquitoes to feed on an infectious blood meal. Infectious blood meals were obtained from an infected 5-d-old chick inoculated subcutaneously 3 d earlier with 10^3^ PFU/0.1 ml WNV or a Hemotek membrane feeder (Discovery Workshops, Accrington, UK, hemotek@discoveryworkshops.co.uk) that contained an infected blood meal consisting of one part virus, one part 50% sucrose, and 19 parts defibrinated goose blood (Hema Resource and Supply, http://www.hemaresource.com/). The titer of WNV in the chick was 10^6.1^, and the titer in the feeder blood meals was 10^7.6^ PFU/ml. Mosquitoes, starved for 24–48 h, were exposed to a lightly restrained chick or membrane feeder for ∼1 h. Fully engorged mosquitoes were removed and maintained at 27 °C, high humidity, and with a photoperiod of 16:8 (L:D), until used in experiments.

Because only ∼50% of *Cx. tarsalis* females orally exposed to WNV become infected, we screened mosquitoes for the presence of a disseminated infection prior to use in experiments. On days 13–14 post-feeding, mosquitoes were anesthetized with CO_2_ and wet ice; one metathoracic leg was removed and placed into a microcentrifuge tube with a BB (Daisy Zinc Plated BB, Rogers, Arkansas, United States) and 1 ml of mosquito diluent (20% heat-inactivated fetal bovine serum in Dulbecco's prosphate-buffered saliva plus 50 ug/ml penicillin/streptomycin, 50 ug/ml gentamicin, and 2.5 μl/ml fungizone). Legs were homogenized in a mixer mill (QIAGEN, http://www.qiagen.com/) at 24 cycles/s for 30 s and then clarified by centrifugation. Virus was detected in clarified homogenate by plaque assay on Vero cells. Presence of virus in the leg indicated a disseminated infection.

### Efficiency of virus recovery from mouse tissues.

To determine the efficiency of virus recovery from mouse tissues, we inoculated a known amount of virus (10^5^ PFU in 1 μl) subcutaneously into the tail (∼2 cm from tip) and ear (∼1 cm from base) for each of three deeply anesthetized C3H mice, using a 30G needle and 100-μl glass syringe (Hamilton, http://www.hamiltoncompany.com/). Immediately following inoculation, the tail and ear were cut off at the base, and the mouse was euthanized. The tail was further divided into 1-cm sections, starting at the tip. Each tissue (or tissue section) was placed into an individual microcentrifuge tube containing 500 μl of BA-1 diluent (M199H, 1% bovine serum albumin, 0.05 M Tris [pH 7.6], 0.35 g/l sodium bicarbonate, 100 U/ml penicillin, 100 μg/ml streptomycin, 1 μg/ml fungizone). As a control, virus (10^5^ PFU in 1 μl) was inoculated directly into each of five microcentrifuge tubes containing 500 μl of BA-1 diluent, using the same needle and syringe. All samples (tissues and controls) were then processed as described below.

### Amount of virus inoculated into host tissue by mosquitoes.

We determined the amount of WNV inoculated by infected female mosquitoes while probing and feeding on a chick toe, mouse tail, or mouse ear. Individual mosquitoes, infected with WNV as described above, were placed into clear plastic 18.5-ml vials with mesh top and starved for ∼48 h prior to feeding. Prior to mosquito feeding, mice were lightly anesthetized with 0.5 mg/g of Avertin (2,2,2 tribromoethanol; Sigma-Aldrich, http://www.sigmaaldrich.com/), and the tail was marked every 1 cm, starting from the tip, with a laboratory marker. Chicks were restrained by hand, and the center toe of the left foot was marked every 0.5 cm starting from the tip. The mesh top of a vial containing one mosquito was placed in contact with the ventral side of the chick toe, ventral side of the mouse tail, or distal half of the mouse ear. Mosquitoes were observed throughout the experiment with a 5× handheld magnifying glass. Probing time, feeding time, blood engorgement status, and probing location (on the tail or toe) were recorded for each mosquito. Blood engorgement status was scored with a range from 1 (blood just able to be detected in abdomen) to 4 (fully engorged). Probing was defined as the period from when the stylets penetrated the skin and the labial sheath folded back, to the first appearance of blood in the abdomen. Feeding began when blood was first detected in the abdomen and ended when a mosquito withdrew her mouthparts from the host. We limited the probing period for each mosquito to a maximum of 10–11 min to minimize the entry of virus into host cells. Immediately after the cessation of feeding or the 10–11 min probing period, the host animal was deeply anesthetized, and the tissue that had been fed upon was cut off at the base and divided into sections (for the tail and toe) with a scalpel. A sample of blood was taken from the wing vein (chicks) or the heart (mice). Animals were then euthanized by cervical dislocation. Average time between cessation of mosquito probing or feeding and tissue excision was 5 min. Tissue and blood samples were held at 4 °C or on wet ice for up to 3 h and then frozen at −80 °C. Prior to freezing, blood samples were centrifuged at 8,000 rpm for 5 min and serum was collected. Tissue samples were processed as described below. Mosquitoes that had fed on or probed the host were killed by freezing, and the legs and body of each mosquito were dissected, placed into separate microcentrifuge tubes containing 1 ml mosquito diluent and a BB, and frozen at −80 °C. Body and leg samples were homogenized and clarified as described above. Virus was quantified in clarified mosquito homogenate by plaque assay on Vero cells. Individual host animals were included in the analysis only if the mosquito that had fed on the animal was subsequently confirmed as positive for viral dissemination (i.e., virus-positive legs).

### Effect of probing time on viral dose inoculated by mosquitoes.

We sought to determine whether longer probing times led to higher inoculated doses of WNV. *Cx. tarsalis* females were infected with WNV by intrathoracic inoculation as described above. At 5 d PI, individual mosquitoes were placed into 18.5-ml clear plastic vials and starved of sucrose and water for 48 h. At 7 d PI, individual mosquitoes were allowed to probe on the tails of lightly anesthetized 12-wk-old C3H female mice for 30 s, 1 min, 2 min, 4 min, or maximum time (*n* = 5/group). Mosquitoes in the maximum time group were able to imbibe blood; mosquitoes in all other groups probed only. Mosquitoes were observed throughout the experiment with a 5× handheld magnifying glass. Feeding time, blood engorgement status, and probing location were recorded for each mosquito as described above. Host tail tissue and blood were harvested as described above.

### Comparison of in vivo and in vitro estimates of viral dose inoculated by mosquitoes.

To compare in vivo and in vitro estimates of the amount of WNV inoculated by mosquitoes, we performed an in vitro capillary tube transmission assay on mosquitoes that had probed and fed on a mouse tail. *Cx. tarsalis* females were infected with WNV by intrathoracic inoculation of ∼300 PFU. At 5 d PI, individual mosquitoes were placed into 18.5-ml clear plastic vials and starved of sucrose and water for 48 h. At 7 d PI, individual mosquitoes were allowed to probe and feed on the tails of lightly anesthetized C3H female mice until they had taken a full blood meal. Mosquitoes were observed throughout the experiment with a 5× handheld magnifying glass. Feeding time, blood engorgement status, and probing location were recorded for each mosquito as described above. Host tail tissue and blood were harvested as described above. Within 2–4 h of host tail feeding, in vitro capillary tube transmission assays were performed using the mosquitoes that had fed on the tails and also those in the same cohort that had been starved, and not allowed to feed. For these assays, each mosquito was anesthetized with triethylamine (Sigma-Aldrich), its legs were removed, and its proboscis was placed into a glass capillary tube filled with a 1:1 solution of 50% sucrose and fetal bovine serum. After 30–40 min, the mosquito was removed from the capillary tube and contents of the capillary tube were expelled into 300 μl of mosquito diluent and frozen at −80 °C until assayed for WNV by plaque assay.

### Tissue sample processing.

Tissue samples and virus controls were thawed on wet ice and poured into a plastic weighing boat. Tissues were macerated with a sterile scalpel. Macerated tissues were returned to their original vial, and 500 μl of BA-1 were added to the weighing boat so as to wash any remaining tissue back into the vial. A BB was added to the vial, and the sample was homogenized in a mixer mill at 24 cycles/s for 8 min and centrifuged at 14,000 rpm for 3 min. Virus was quantified in clarified tissue homogenate, control samples, and sera by plaque assay on Vero cells.

### WNV titer in mosquito body segments.

The distribution of WNV in mosquito body segments was determined. Orally or parenterally infected *Cx. tarsalis*, and parenterally infected *Cx. pipiens* were killed by freezing at −80 °C at 16 or 7 d post-infection, respectively. Mosquitoes were later thawed on wet ice, their legs were removed, and the head, thorax, and abdomen were cut apart with a scalpel. Body segments were placed into separate microcentrifuge vials containing 1 ml of mosquito diluent and a BB. Tubes containing mosquito body parts were homogenized as described above. Virus was detected in clarified mosquito homogenate by plaque assay on Vero cells.

### Clearance of virus from blood following intravenous inoculation.

Four adult C57/BL6 mice were inoculated intravenously in the lateral tail vein with 10^5^ PFU in a volume of 0.1 ml. Blood samples were taken at 5, 15, and 45 min PI by tail bleeding. Mice were euthanized at 90 min PI, and a blood sample was taken from the heart. Blood samples were centrifuged at 8,000 rpm for 5 min, and serum was collected and frozen at −80 °C until tested for virus by plaque assay on Vero cells.

### Statistical analysis.

Viral titers were log transformed and checked for normality using Shapiro-Wilk or Kolmogorov-Smirnov statistics. The limits of detection for plaque assays were 5 PFU for mosquito, mouse, and chick tissues, and 5 PFU/ml for serum. Nonparametric tests were used when we compared groups with small sample sizes or non-normal distributions. One-way ANOVA was used to test for differences between virus titers in mosquito body segments; the Tukey-Kramer method was used to adjust for multiple comparisons. Viral clearance data was normalized to the 5-min titer, and Graph Pad Prism software (http://www.graphpad.com/) was used to fit a linear regression model.

## Supporting Information

Table S1 Amount of WNV Inoculated by Parenterally Infected *Cx. tarsalis* while Probing and Feeding on a Mouse Tail (57 KB DOC)

Table S2 Amount of WNV Inoculated by Orally Infected *Cx. tarsalis* while Probing and Feeding on a Mouse Tail (54 KB DOC)

Table S3 Amount of WNV Inoculated by Orally and Parenterally Infected *Cx. tarsalis* while Probing and Feeding on a Mouse Ear (37 KB DOC)

Table S4 Amount of WNV Inoculated by Parenterally Infected *Cx. tarsalis* while Probing and Feeding on a Chick Toe (41 KB DOC)

Table S5 Amount of WNV Inoculated by Parenterally Infected *Cx. pipiens* while Probing and Feeding on a Chick Toe (41 KB DOC)

Table S6 Amount of WNV Inoculated by Parenterally Infected *Ae. japonicus* while Probing and Feeding on a Mouse Tail (41 KB DOC)

Table S7 Amount of WNV Inoculated by Parenterally Infected *Ae. triseriatus* while Probing and Feeding on a Mouse Tail (37 KB DOC)

## Abbreviations

LOD: limit of detection
PFU: plaque forming units
PI: post-inoculation
VEEV: Venezuelan equine encephalitis virus
WNV: West Nile virus
