# Supplementary material for: Mosquitoes Inoculate High Doses of West Nile Virus as They Probe and Feed on Live Hosts
Source: PLoS Pathog. 2007 Sep 14;3(9):e132. doi: 10.1371/journal.ppat.0030132 (PMC1976553; doi:10.1371/journal.ppat.0030132)
Supplement: Supplementary file 2 [file ppat.0030132.st002.doc]

Table S2. Amount of WNV inoculated by orally infected *Cx. tarsalis* while probing and feeding on a mouse tail.

| **Mouse** | **log_10_ PFU in tail section** | | | | | | | | **Probe** | **Feed** | **Engorgement** | **Serum titer** |
| --- | --- | --- | --- | --- | --- | --- | --- | --- | --- | --- | --- | --- |
| **ID #** | **1 (tip)** | **2** | **3** | **4** | **5** | **6** | **7** | **Total** | **time (min)** | **time (min)** | **status** | **(log_10_ PFU/ml)** |
| 7-5 | 0 | 1.0 | 5.3 | 1.7 | 0.7 | 0 | 0 | 5.3 | 5 | 5 | 4 | 2.7 |
| 7-6 | 0 | 0 | 1.7 | 5.2 | 1.3 | 1.2 | 0.7 | 5.2 | 10 | NA | 0 | 0 |
| 7-1 | 0 | 1.3 | 4.5 | 1.6 | 1.2 | 0 | 0 | 4.5 | 10 | 1 | 1 | 1.5 |
| 7-2 | 0 | 1.0 | 4.3 | 0 | 0 | 0.7 | 0 | 4.3 | 5 | 5 | 4 | 0 |
| 7-9 | 0 | 0 | 4.1 | 2.0 | 1.4 | 1.0 | 0.7 | 4.1 | 10 | NA | 0 | 0 |
| 6-1 | 0 | 3.5 | 2.2 | 1.0 | 0 | 0 | 0 | 3.5 | 3 | 3 | 4 | 1.4 |
| 7-3 | 0 | 0 | 3.4 | 1.0 | 0 | 0.7 | 0 | 3.4 | 8 | 2 | 4 | 1.6 |
| 7-11 | 0 | 0 | 0 | 3.2 | 0 | 0 | 0 | 3.2 | 7 | 3 | 3 | 1.0 |
| 7-4 | 0 | 0 | 2.4 | 0 | 0 | 0 | 0 | 2.4 | 5 | NA | 0 | 0 |
| 7-10 | 0 | 0 | 0 | 0 | 0 | 0 | 0 | 0 | 5 | 1 | 4 | 0 |
|  |  |  |  |  | **Mean** | | | **3.6** | **6.8** | **2.9** | **-** | **0.8** |
|  |  |  |  |  |  | **Median** | | **3.8** | **6.0** | **3.0** | **-** | **0.5** |

Shaded cells in the table indicate sections of the tail in which the mosquito probed or fed. Individual host animals were included in the study only if the mosquito that fed on the animal was confirmed as positive for viral dissemination. LOD for plaque assay was 5 PFU for tissue and 5 PFU/ml for serum. Values less than LOD are reported as 0.

NA: Mosquitoes did not imbibe blood from tissue.

Engorgement status: 0=did not feed, 1=blood just detectable in abdomen (0-25% full), 2= 25-50% full, 3=50-75% full, 4=completely engorged (75-100% full).
