# Supplementary material for: Mosquitoes Inoculate High Doses of West Nile Virus as They Probe and Feed on Live Hosts
Source: PLoS Pathog. 2007 Sep 14;3(9):e132. doi: 10.1371/journal.ppat.0030132 (PMC1976553; doi:10.1371/journal.ppat.0030132)
Supplement: Supplementary file 3 [file ppat.0030132.st003.doc]

Table S3. Amount of WNV inoculated by orally and parenterally infected *Cx. tarsalis* while probing and feeding on a mouse ear.

| **Mouse** | **Mosquito infection** | **Total titer** | **Probe** | **Feed** | **Engorgement** | **Serum titer** |
| --- | --- | --- | --- | --- | --- | --- |
| **ID #** | **method** | **(log_10_ PFU)** | **time (min)** | **time (min)** | **status** | **(log_10_ PFU/ml)** |
| 4-3 | IT Inoc | 5.4 | 10 | NA | 0 | 0 |
| 7-7 | Oral | 5.3 | 9 | NA | 0 | 0 |
| 5-1 | IT Inoc | 5.0 | 2 | 5 | 3 | 1.0 |
| 4-4 | IT Inoc | 4.7 | 7 | 8 | 1 | 1.5 |
| 4-5 | IT Inoc | 4.5 | 6 | NA | 0 | 0 |
| 7-8 | Oral | 4.1 | 7 | NA | 0 | 0 |
|  | **Mean** | **4.8** | **6.8** | **6.5** | **-** | **0.4** |
|  | **Median** | **4.9** | **7.0** | **6.5** | **-** | **0.0** |

IT Inoc = intrathoracic inoculation of ~30 PFU.

NA: Mosquitoes did not imbibe blood from tissue.

LOD of plaque assay was 5 PFU/ml for serum. Values less than LOD are reported as 0.

Engorgement status: 0=did not feed, 1=blood just detectable in abdomen (0-25% full), 2= 25-50% full, 3=50-75% full, 4=completely engorged (75-100% full).
