# Supplementary material for: Mosquitoes Inoculate High Doses of West Nile Virus as They Probe and Feed on Live Hosts
Source: PLoS Pathog. 2007 Sep 14;3(9):e132. doi: 10.1371/journal.ppat.0030132 (PMC1976553; doi:10.1371/journal.ppat.0030132)
Supplement: Supplementary file 4 [file ppat.0030132.st004.doc]

Table S4. Amount of WNV inoculated by parenterally infected *Cx. tarsalis* while probing and feeding on a chick toe.

| **Chick** | **log_10_ PFU in toe section** | | | | | **Probe** | **Feed** | **Engorgement** | **Serum titer** |
| --- | --- | --- | --- | --- | --- | --- | --- | --- | --- |
| **ID #** | **1 (tip)** | **2** | **3** | **4** | **Total** | **time (min)** | **time (min)** | **status** | **(log_10_ PFU/ml)** |
| 3-4 | 5.6 | 4.7 | 3.0 | 4.1 | 5.7 | 6 | 1 | 1 | 3.7 |
| 3-1 | 5.5 | 3.5 | 2.8 | 2.8 | 5.5 | 7 | 4 | 4 | 3.1 |
| 1-1 | 4.3 | 4.0 | 0 | 0 | 4.5 | 7 | NA | 0 | 0 |
| 1-2 | 4.4 | 2.5 | 1.7 | 1.3 | 4.4 | 5 | NA | 0 | 0 |
| 3-2 | 1.3 | 3.1 | 1.0 | 0.7 | 3.1 | 3 | NA | 0 | 0 |
| 3-3 | 1.0 | 0 | 0 | 0 | 1.0 | 3 | 4 | 4 | 2.0 |
| 1-3 | 0.7 | 0 | 0 | 0 | 0.7 | 3 | NA | 0 | 0 |
|  | **Mean** | | | | **3.6** | **4.9** | **3.0** | **-** | **1.3** |
|  |  |  | **Median** | | **4.4** | **5.0** | **4.0** | **-** | **0.0** |

Shaded cells in the table indicate sections of the toe in which the mosquito probed or fed. NA: Mosquitoes did not imbibe blood from tissue. LOD for plaque assay was 5 PFU for tissue and 5 PFU/ml for serum. Values less than LOD are reported as 0.

Engorgement status: 0=did not feed, 1=blood just detectable in abdomen (0-25% full), 2= 25-50% full, 3=50-75% full, 4=completely engorged (75-100% full).
