# Supplementary material for: Mosquitoes Inoculate High Doses of West Nile Virus as They Probe and Feed on Live Hosts
Source: PLoS Pathog. 2007 Sep 14;3(9):e132. doi: 10.1371/journal.ppat.0030132 (PMC1976553; doi:10.1371/journal.ppat.0030132)
Supplement: Supplementary file 5 [file ppat.0030132.st005.doc]

Table S5. Amount of WNV inoculated by parenterally infected *Cx. pipiens* while probing and feeding on a chick toe.

| **Chick** | **log_10_ PFU in toe section** | | | | | **Probe** | **Feed** | **Engorgement** | **Serum titer** |
| --- | --- | --- | --- | --- | --- | --- | --- | --- | --- |
| **ID #** | **1 (tip)** | **2** | **3** | **4** | **Total** | **time (min)** | **time (min)** | **status** | **(log_10_ PFU/ml)** |
| 3-7 | 6.6 | 3.8 | 3.1 | 3.4 | 6.6 | 3 | 1 | 1 | 3.9 |
| 3-6 | 5.7 | 3.2 | 1.6 | 3.3 | 5.7 | 4 | NA | 0 | 2.7 |
| 3-10 | 5.7 | 0 | 0 | 0 | 5.7 | 2 | NA | 0 | 0 |
| 3-5 | 3.6 | 5.6 | 0 | 0 | 5.6 | 3 | NA | 0 | 0 |
| 1-4 | 5.2 | 2.4 | 1.7 | 2.0 | 5.2 | 6 | 1 | 2 | 2.3 |
| 3-8 | 5.0 | 0 | 0 | 0 | 5.0 | 5 | NA | 0 | 0 |
| 3-9 | 4.1 | 0 | 0 | 0 | 4.1 | 3 | NA | 0 | 0 |
|  | **Mean** | | | | **5.4** | **3.7** | **1.0** | **-** | **1.3** |
|  |  |  | **Median** | | **5.6** | **3.0** | **1.0** | **-** | **0.0** |

Shaded cells in the table indicate sections of the toe in which the mosquito probed or fed. NA: Mosquitoes did not imbibe blood from tissue. LOD for plaque assay was 5 PFU for tissue and 5 PFU/ml for serum. Values less than LOD are reported as 0.

Engorgement status: 0=did not feed, 1=blood just detectable in abdomen (0-25% full), 2= 25-50% full, 3=50-75% full, 4=completely engorged (75-100% full).
