# Supplementary material for: Mosquitoes Inoculate High Doses of West Nile Virus as They Probe and Feed on Live Hosts
Source: PLoS Pathog. 2007 Sep 14;3(9):e132. doi: 10.1371/journal.ppat.0030132 (PMC1976553; doi:10.1371/journal.ppat.0030132)
Supplement: Supplementary file 7 [file ppat.0030132.st007.doc]

Table S7. Amount of WNV inoculated by parenterally infected *Ae. triseriatus* while probing and feeding on a mouse tail.

| **Mouse** | **log_10_ PFU in tail section** | | | | | | | | **Probe** | **Feed** | **Engorgement** | **Blood titer** |
| --- | --- | --- | --- | --- | --- | --- | --- | --- | --- | --- | --- | --- |
| **ID #** | **1 (tip)** | **2** | **3** | **4** | **5** | **6** | **7** | **Total** | **time (min)** | **time (min)** | **status** | **(log_10_ PFU/ml)** |
| 8-7 | 0 | 0 | 0 | 0 | 3.3 | 4.0 | 1.2 | 4.1 | 1 | 1 | 1 | 1.3 |
| 8-6 | 0 | 0 | 0 | 2.9 | 0 | 0 | 0 | 2.9 | 3 | 1 | 4 | 0 |
| 8-8 | 0 | 0 | 0 | 1.2 | 2.3 | 0 | 0 | 2.4 | 3 | 1 | 1 | 0 |
|  |  |  |  |  |  |  | **Mean** | **3.1** | **2.3** | **1.0** | **-** | **0.4** |
|  |  |  |  |  |  |  | **Median** | **2.9** | **3.0** | **1.0** | **-** | **0.0** |

Shaded cells in the table indicate sections of the tail in which the mosquito probed or fed. LOD for plaque assay was 5 PFU for tissue and 5 PFU/ml for serum. Values less than the LOD are reported as 0.

NA: Mosquitoes did not imbibe blood from tissue.

Engorgement status: 0=did not feed, 1=blood just detectable in abdomen (0-25% full), 2= 25-50% full, 3=50-75% full, 4=completely engorged (75-100% full).
